# Social visual attention as a treatment outcome: evaluating the social games for autistic adolescents (SAGA) intervention

**DOI:** 10.1038/s41598-024-51332-z

**Published:** 2024-01-05

**Authors:** K. Suzanne Scherf, Jason W. Griffin, Charles F. Geier, Joshua M. Smyth

**Affiliations:** 1 Department of Psychology, Pennsylvania State University, 113 Moore Building, University Park, 16802 USA; 2 Department of Human Development and Family Studies, University of Georgia, Athens, USA; 3 Department of Psychology, Ohio State University, Columbus, USA

**Keywords:** Human behaviour, Autism spectrum disorders

## Abstract

A core feature of autism involves difficulty perceiving and interpreting eye gaze shifts as nonverbal communicative signals. A hypothesis about the origins of this phenotype is that it emerges from developmentally different social visual attention (SVA). We developed Social Games for Autistic Adolescents (SAGA; Scherf et al. BMJ Open 8(9):e023682, 2018) as a serious game intervention for autistic individuals to discover the significance of eye gaze cues. Previously, we demonstrated the effectiveness of SAGA to improve the perception and understanding of eye gaze cues and social skills for autistic adolescents (Griffin et al. JCPP Adv 1(3):e12041, 2021). Here, we determine whether increases in social visual attention to faces and/or target gazed-at objects, as measured via eye tracking during the same Gaze Perception task in the same study sample, moderated this improvement. In contrast to predictions, SVA to faces did not differentially increase for the treatment group. Instead, both groups evinced a small increase in SVA to faces over time. Second, Prior to the SAGA intervention, attention to faces failed to predict performance in the Gaze Perception task for both the treatment and standard care control groups. However, at post-test, autistic adolescents in the treatment group were more likely to identify the object of directed gaze when they attended longer to faces and longer to target objects. Importantly, this is the first study to measure social visual attention via eye tracking as a *treatment response* in an RCT for autism. NCT02968225

## Introduction

Autism spectrum disorder (ASD) is a lifetime condition that impacts social communicative behavior^1^. A core feature of autism is difficulty perceiving and interpreting shifts in eye gaze as nonverbal communicative signals. These shifts in gaze are used to communicate the relative importance of objects and people in the world. Developing sensitivity to eye gaze shifts is foundational to multiple social communicative sills, including joint attention^2^, language^3^, and theory of mind^4^. Difficulties processing shifts in eye gaze are present in infants later diagnosed with ASD^5^ and persist for autistic individuals through childhood^6^, adolescence^7^ and adulthood^8^. These difficulties are largely related to understanding the referential nature of gaze and/or assigning social relevance to gazed-at objects^9–11^. As a result, autistic individuals are likely missing critical non-verbal social cues that contextualize and direct social interactions in a variety of social contexts.

An influential hypothesis about the origins of this core autism phenotype is that it emerges from developmentally different social visual attention, particularly to faces^12–14^. In other words, infants and children on the autism spectrum do not attend to faces in the same way or to the same extent required to learn about the functional utility of many aspects of nonverbal communicative signaling via the face, including shifts in eye gaze. This different developmental context could lead to a state in which the face and “eyes are not meaningful” to autistic individuals^15^. The implication from this hypothesis is that it may be possible to create opportunities for autistic individuals to discover the functional significance of eye gaze cues by developing effective methods to encourage them to focus visual attention on faces. In turn, this may provide novel learning contexts and potentially alter multiple aspects of social functioning, like face processing and social communication.

### Social games for autistic adolescents (SAGA) psychosocial intervention

This was the central goal motivating the development of the SAGA intervention^16,17^. SAGA is a psychosocial intervention (i.e., non-pharmacological treatment aimed at improving the functioning of individuals in any of the impairments characteristic of ASD^18^) that was developed using the *experimental therapeutics* approach^19^. It was specifically designed to increase sensitivity to shifts in eye gaze as non-verbal social communicative cues, in part, by scaffolding visual attention to faces. It is important to note that the goal of this intervention is to support implicit learning about how eye gaze cues are used as nonverbal social cues to guide and contextualize social interactions. It is very similar to the goal of helping individuals on the spectrum learn to perceive and interpret facial expressions^20^. Such learning can potentially provide autistic individuals with important information about the actions and intentions of others, which can facilitate their own decisions about behaving in social interactions. Importantly, the intervention does not force participants to employ a particular social communicative behavior (e.g., look at faces or eyes). It only provides *opportunities for learning* in a safe environment. It is an open question whether participants will learn the information and go on to use it to inform social communicative behaviors in real world social interactions.

It is a “serious game”^21^ delivered on a computer in the home. Serious games are intervention tools designed to improve targeted skills (including those that are difficult and not intrinsically rewarding for participants), with the goal of enhancing real life outcomes^21^. They integrate educational objectives with evidence-based game mechanics known to support intrinsic motivation and generalization of learning outside of the game context. The SAGA intervention is designed to scaffold learning about the referential understanding of eye gaze cues in simulated social interactions with computer-animated human characters, which are embedded in an age-appropriate narrative storyline^16^. Players must discover that eye gaze cues can be used to guide their own goal-directed behavior to solve problems in the game. This simulates the way eye gaze cues are discovered developmentally and used in the real world.

The serious game mechanics there were employed in the design of SAGA are described in detail in^16^. Most of these mechanics foster intrinsic motivation for sustainable and generalizable learning. They include an immersive storyline (i.e., pet detective looking for lost pet) with clear goals (e.g., make a potion to dissolve the gum on the school locker to see if there is a clue about where the pet is), rewards and feedback specifically about goals progress (i.e., gum is nearly dissolved, need to make more potion to finish the job), increasing levels of difficulty (e.g., learn about how gaze provides information about locations and objects in the world before learning how to engage in joint attention), individualized training (i.e., continuously changing difficulty to optimize performance at ~ 80%), and the provision of choice (e.g., participants always presented with choices).

We designed SAGA for adolescents on the autism spectrum for several reasons. First, adolescence is a potentially vulnerable period of development in ASD when developmental trajectories decline or plateau, especially regarding the processing of social information, making it an important time for intervention (for review see^22,23^). This is evident when adolescence is defined more narrowly (e.g., the second decade of life—World Health Organization) and when it is defined more broadly to include emerging adulthood (~ age 10–25 years^24^). Second, social looking behavior may change in important ways during adolescence that require a new attentional focus on faces and eye gaze as individuals transition into new social roles with their peers. Third, adolescents are old enough to tolerate a protocol that involves several hours of training each week over the course of 10 weeks, which is the minimal amount of training we hypothesized would be required to observe emerging changes in sensitivity to eye gaze cues.

In a small-scale randomized clinical trial (RCT), we investigated the initial effectiveness of SAGA^17^. Autistic adolescents were randomized into either a treatment or standard care control condition. Participants in the treatment group were asked to play SAGA for 30-min sessions at home 3 times a week over 10 weeks. Following the intervention, the treatment group improved their understanding that an observer’s visual behavior is directed towards objects in the world and that it involves the mental experience of seeing something^17^. This learning enabled them to use eye gaze cues to guide their own goal-directed social interactions with human-like avatars. The learning also generalized to enable the autistic adolescents to identify directed gaze behavior more accurately in *human* faces. There was some evidence of a dose-dependent response; participants in the treatment group who received a sufficient dose of gameplay showed larger treatment-related improvements. Finally, increases in sensitivity to human eye gaze cues were associated with improvements in parent-reported social skills. Given this evidence of improved sensitivity to the non-verbal communicative nature of eye gaze cues, it is important to evaluate whether *social visual attention was a moderating mechanism* of this improvement.

### Current study

In the present work, we investigated whether this improved behavioral sensitivity to eye gaze cues was facilitated by *increasing visual attention to faces and/or to the target objects* of the directed gaze. Specifically, we included both primary outcomes measures (behavioral accuracy, eye tracking metrics of social visual attention) from the original RCT to understand whether SAGA effectively altered social visual attention in addition to sensitivity to eye gaze cues. Here, we present the so far unpublished eye-tracking data from the original participants in combination with the previously published behavioral data^17^.

We predicted that if social visual attention is successfully targeted by the SAGA intervention, we would expect to see increasing attention to faces specifically in the treatment group. Additionally, we predicted that if social visual attention moderates behavioral improvements in sensitivity to directed eye gaze in autistic adolescents, then we would observe an emerging association between the accuracy of performance and attention to faces, following the SAGA intervention.

## Method

The protocol for this randomized controlled trial (RCT) is published in its entirety^16^. It is also registered through clinicaltrials.gov (NCT02968225). The study was performed in accordance with all relevant guidelines and regulations. What follows is a brief overview of this extensive protocol.

### Trial design

SAGA was an experimental, two-arm, Phase 1/2 RCT. Autistic adolescents were randomized to receive the serious game training or to continue standard care. This study was approved by the Institutional Review Board at Pennsylvania State University. An independent board monitored safety and examined interim feasibility and effectiveness results. The study protocol is published elsewhere^16^. Both sensitivity to eye gaze cues, as measured by behavioral accuracy, and social visual attention to faces and gazed-at objects, as measured by eye tracking, were preregistered as primary outcome measures^16^ (see ClinicalTrials.gov NCT02968225). Both these outcome measures from the *baseline session* were published to compare performance of autistic adolescents with that of age-, sex-, and IQ-matched non-autistic adolescents^11^. The initial evaluation of SAGA effectiveness was reported using the sensitivity to eye gaze cues measure (i.e., behavioral data)^17^. The effectiveness of SAGA using social visual attention (i.e., eye tracking data) has not been reported. Here, we present findings to evaluate the effectiveness of SAGA using the social visual attention outcome measure in the same RCT sample. The eye tracking data will be available in the NIMH National Data Archive.

### Participants and trial procedures

#### Recruitment

Adolescents, ages 10–18 years, and their families were recruited from two research databases: Interactive Autism Network Research Database at the Kennedy Krieger Institute, Baltimore, and autismMatch at the Center for Autism Research, Philadelphia. Initial eligibility was assessed online or by phone^16^. Full eligibility was assessed in the Laboratory of Developmental Neuroscience at Pennsylvania State University (PSU). Participants were enrolled between December 2017 and February 2018 and followed up between March 2018 and May 2018.

#### Inclusion/exclusion criteria^16^

Participants had to (1) have a diagnosis of ASD confirmed by the ADOS-2^25^; (2) be a native English speaker; (3) be 10–18 years of age; (4) have normal vision and hearing with correction; (5) be able to use a computer; (6) score < 80% correct on an eye gaze screening task; (7) have a full scale IQ of 70–130, assessed using the KBIT-2^26^; and (8) have at least a second grade reading level, assessed by the OWLS-2^27^. Participants were excluded if they (1) had seizures within previous 2 years; (2) lacked stable home internet; (3) parent or adolescent refused to consent/assent, (4) adolescent was 18 and had a legal guardian, prohibiting him/her from legally consenting, or (5) adolescent was 18 and could not understand the consent.

Written informed consent was obtained from parents of participants and 18-year-old adolescents and written assent was acquired from the adolescents aged 10–17 years to participate in the study according to procedures approved by the Institutional Review Board (IRB) at Penn State University. Eligibility assessments were only administered after both consent and assent were obtained. Participants who met the final eligibility criteria were invited to continue with the pre-intervention testing session.

#### Randomization

After consent and pre-intervention assessments, participants were randomly assigned to 1 of 2 conditions (serious game treatment vs standard care control) over a period of 10 weeks by the Principal Investigator (KSS) who did not have any involvement with clinical or experimental procedures. Randomization proceeded according to a computer-generated list in a 1:1 ratio (serious game treatment, standard care), stratified by gender and full-scale IQ (> 100, < 100).

#### Blinding procedures

Parents and adolescent participants were not blinded from knowing the condition assignment. However, they were also not told that the intervention was targeting eye gaze cues. Participants and parents were told that the project was designed to learn about how “autistic adolescents may learn social skills from playing a computer game,” and adolescents are instructed to “interact with animated characters and recognize nonverbal social cues, such as pointing, head turns, or eye gaze cues.” Therefore, the focus on social attention to faces and on understanding eye gaze cues was never explicitly described to participants or parents.

Researchers involved in the clinical assessment and data collection procedures were blinded from condition assignment during the preintervention data collection session. Researchers involved in ensuring the fidelity of the intervention were not involved in the data collection procedures. Researchers involved in the data collection at the post-intervention visit were blinded from condition assignment as well. However, in some cases unblinding occurred when participants volunteered information about their experience in the intervention during this visit. Importantly, the primary outcome measures are believed to be robust to investigator bias.

### Measures and assessments

#### SAGA: a serious game intervention

SAGA is an adventure game with embedded serious game techniques in which participants solve problems in a 3D environment that is programmed in Unity (https://unity3d.com/unity). The core training mechanisms are delivered via character interactions. Participants learn skills through simulated social interactions with human avatars in the game as they solve problems related to the game narrative (i.e., rescuing a lost pet as a Ped Detective). Each social scene has variable elements, including different objects, locations, and levels of difficulty, that are dynamically altered. Moreover, scenes are presented with a variety of characters and environmental contexts to enhance engagement and support generalized learning opportunities.

The game is designed to train learning about three functional uses of eye gaze cues, including the use of gaze to reference locations and objects in the world via a single informant and in episodes of joint attention between multiple informants. Three sequential phases are implemented in the game. Tasks in phase 1 are structured to help participants learn that eye gaze is an important cue to solving problems in the game. Tasks in phase 2 help participants learn to estimate precise gaze trajectories by making potential gazed-at objects closer together and to ignore salient objects that are not the locus of the gaze cue. Episodes of joint attention are also introduced in phase 2 as participants must determine the target object that two avatars are looking at together. This is difficult because the timing of the non-verbal cues to identify the object is not perfectly synchronous between the two avatars. In phase 3, tasks are structured around helping participants learn the difference between a goal-directed gaze cue (e.g., looking at a target object to solve a puzzle) and a non-goal-directed gaze cue (e.g., looking around at all the objects before deciding which one to select).

To complete a phase of the game, participants must finish all levels within a phase. Each phase has multiple levels. Levels are defined by the number of non-verbal cues (e.g., hand pointing, shoulder and head orientation, eye gaze cues) avatars use to guide participants to solve puzzles in the game. Easy levels have multiple cues and the progression to subsequent levels increasingly focuses learning to use eye gaze cues exclusively by stripping away other cues. Within each level, there are six stages. Each stage represents the number of potential objects or locations that the participant must discriminate between based on the gaze cue from the avatar. In the easiest stage (i.e., Stage 1), the participant chooses between two objects or locations that the avatar is pointing, directing shoulders, head, and gaze to, whereas in Stage 6, the participant chooses between six possible objects or locations that the avatar could be referring to with the non-verbal cue(s). Within each stage, participants must perform with 80% accuracy to advance to the next stage, and they must finish all stages within a level before they can progress to the next level within a phase. When they perform < 80% accuracy within a stage, they return to the previous stage to reify learning where they were recently successful (including, if necessary, returning to later stages of previous levels). Comprehensive details about the game design and learning mechanisms are explained elsewhere^16^.

The treatment group was instructed to download the SAGA game on their own computer and play it at home for three 30-min sessions per week for 10 weeks. To improve adherence, research staff helped families to identify a potential schedule of game play during the intervention period that included specific days of the week and approximate times of day (e.g., Mondays, Wednesdays, Fridays after school). Families chose whether they wanted to receive reminder texts on these days that said, “PSU Autism Intervention: This is your day to play the game! For help visit http://sagaintervention.com/welcome.html.” If they were receiving text reminders, families also received encouragement texts following play sessions that said, “PSU Autism Intervention: Well done! You have played 30 min and earned $5.” If participants missed a day of game play, the family was called to discuss whether the participant experienced technical difficulties with the game, which could then be addressed, and/or had difficulty fitting game play into their schedule. Research staff were trained to be sensitive to family issues and help the family adjust the training schedule if needed.

To minimize the potential intrusiveness of game play on participants’ schooling and family activities, it was programmed to close after 90 min of play on a single day. This ‘dose’ of treatment was estimated based on the tolerance and relative amount of training required to evince learning in prior face-processing intervention studies in ASD^28^. The goal was for participants to obtain at least 10 h of training specifically on eye gaze tasks across the 10-week training period, which may have required a total of 15–20 h of total game play. Participants could earn up to $200 for game play in the intervention over the 10 weeks.

#### Intervention outcomes

We report the number of sessions played, total number of hours played, and hours engaged in eye gaze tasks.

#### Safety outcomes

A Data Safety Monitoring Board (DSMB) composed of independent researchers who have expertise complementary to the aims of SAGA regularly reviewed the safety and tolerance of the intervention for our participants. Any adverse events were reported to both the DSMB and the Penn State Institutional Review Board.

Potential adverse events and unintended effects occurring during testing were monitored by the research staff. Additionally, self-report and behavioral measures were used to monitor unanticipated risks. This included a Usability questionnaire about the intervention game experience in which participants rated multiple aspects of game play on a Likert scale (e.g., Experience was fun; I felt discouraged) at the post-intervention testing session. As required by the National Institutes of Health, procedures were in place to monitor suicidal ideation and self-injurious behavior among adolescents and to make recommendations about care based on the assessment outcome.

### Primary outcomes

#### Behavior—Gaze perception task

This task was used to evaluate the ability to process and interpret eye gaze cues as in prior studies^7,9,11,29^. In this task, participants view photographs of an actor looking at a single object in a complex scene. Participants are required to identify the gazed-at target object by selecting the item label in a four-alternative-forced-choice (4AFC) task. To successfully identify the gazed-at object, participants must establish a psychological connection between the looker and the content^30^. This allows for *referential understanding* of the observer’s visual behavior; that is, an understanding that visual behavior is directed towards objects/content (i.e., it is not abstract in nature) and that it involves the mental experience of seeing something^2^.

Participants viewed images (4000ms) of actors in a naturalistic setting looking at one of many possible objects. Participants were instructed to identify the object that the person is looking at from a list of four labels presented on a subsequent screen (i.e., multiple choice). Success in this task requires that participants can perceive the trajectory of gaze and understand the referential intent of the actor to look at a specific object. The task included a total of 40 trials at each session (pre, post-testing). In each session, 62.5% (N = 25) images were novel and 37.5% (N = 15) of the images repeated; therefore, a total of 65 images were used from the original dataset^16^. Importantly, of the repeated images, the percentage of agreement at pre- and post-intervention in the standard care control group was 72%, indicating moderate test–retest-reliability^31^.

**Figure 1:**
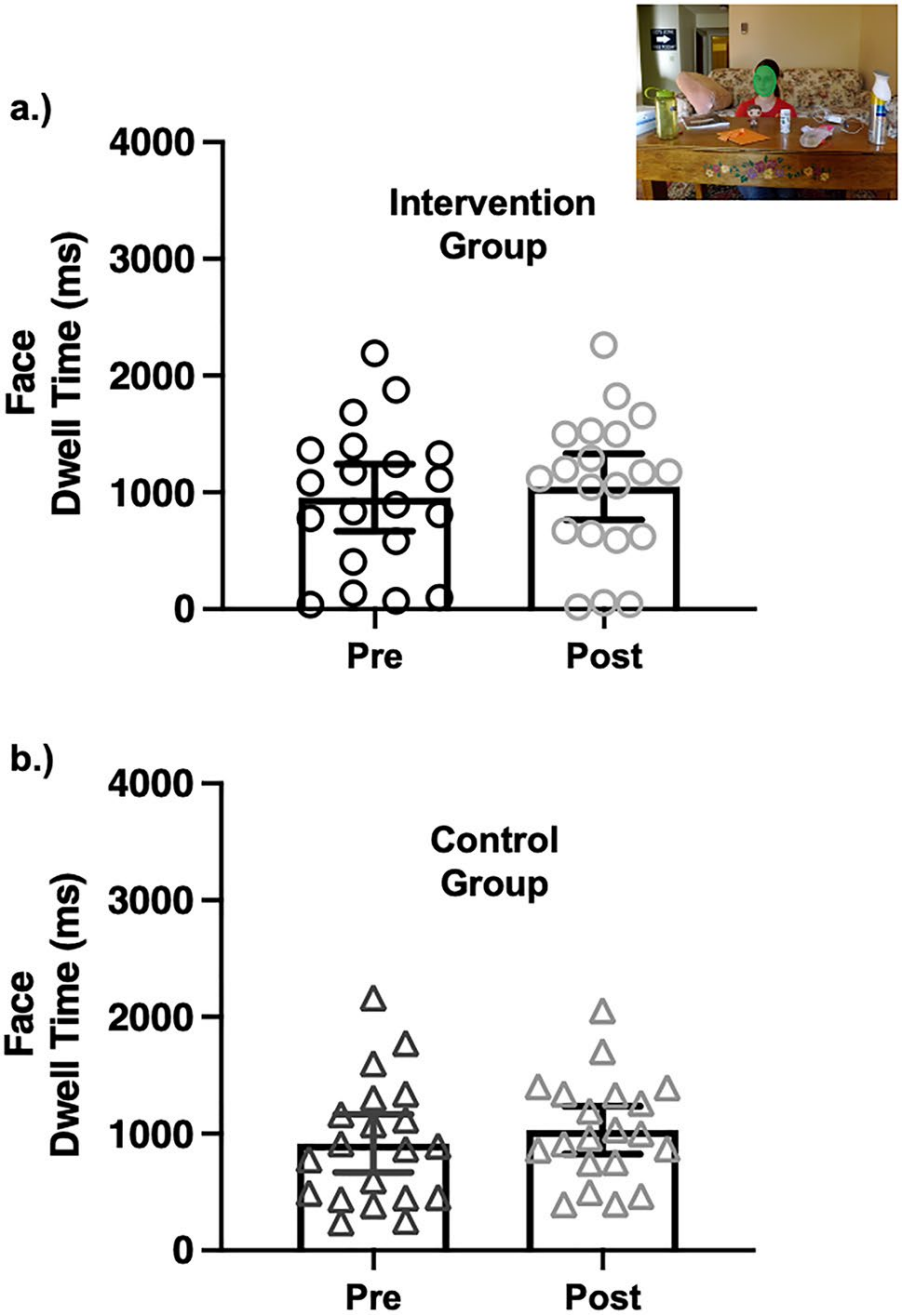
Social visual attention to faces over time. The average dwell time to faces is plotted for each participant as a function of group and session. The individual SAGA intervention participants (**a**) are plotted as circles and the individual standard care control participants (**b**) are plotted as triangles. In contrast to predictions, participating in SAGA did not differentially increase social attention to faces in the intervention group. Instead, both groups evinced a small (~ 100 ms) increase in dwell time to faces at the post testing session. Error bars reflect 95% confidence intervals. All analyses used mixed-effects modeling of trial-level data: however, for visualization these plots reflect mean-level data.

The stimuli were designed such that relying on the general direction of the gaze or of the head (e.g., left of center) would not be sufficient to perform above 50% correct in the task. Specifically, in 50% of the images, the head and gaze cues were misaligned with the actors’ head facing forward and only the eye gaze was directed at the target object (see Fig. 1 in^16^). As a result, simply following head direction was not a reliable cue to identifying the target object more than 50% of the time. Second, on each trial the image contained both the target object and a plausible non-target object (i.e., near the target object but not gazed at). Labels for both these items were presented in the multiple-choice options. Therefore, participants had to estimate the gaze trajectory from the eyes precisely to differentiate the target- and plausible non-target objects and perform correctly more than 50% of the time. The full details about the creation and validation of the stimuli are described elsewhere^32^ and are available in an online repository^33^. This task took approximately 8 min to complete.

#### Eye-tracking

Eye tracking data were collected while the stimulus was displayed on the screen, and not during the presentation of the multiple-choice options. TobiiPro Studio was used for stimuli presentation and eye-tracking data acquisition. Fixation data were collected using a Tobii X2-60 eye tracker (sampling rate of 60 Hz, ~ accuracy of 0.4°, precision of 0.34°) that was integrated with a Dell Optiplex 7040 computer and 24-in. monitor (60 Hz refresh rate). Participants were positioned ~ 65 cm from the display monitor. All stimuli were presented in a fixed order at both time points. A 9-point automatic calibration procedure was used for all participants. Excessive calibration error (> 1°) was re-calibrated for each point.

Raw gaze data were preprocessed using the Tobii identification velocity threshold filter with a minimum fixation duration of 100 ms. Gaze samples were only included when there was recordable information from at least one eye *on the stimulus*. For each participant and stimulus item, the *dwell time* was computed within defined areas of interest (AOIs). Dwell time was the summed total of all fixation durations (in milliseconds) for the entire stimulus within each AOI. AOIs for faces, target objects, and non-target objects were created manually for each stimulus. For face AOIs, we used ellipsoid shapes and positioned them such that the boundaries were at the bottom of the chin, top of the forehead, and between the ears (i.e., excluding hair and ears). For all objects, we manually constructed “hard” boundary AOI’s by creating a polygon shape with vertices at each convex vertex of the object.

### Statistical analysis

The goals of the analyses were two-fold. First, we evaluated whether SAGA differentially improved social visual attention (i.e., dwell time) to faces as a function of participating in the intervention. Second, we investigated whether the SAGA-related improvements in the ability to process and interpret eye gaze cues (i.e., behavioral accuracy) were moderated by increases in visual attention to faces and/or target objects (i.e., dwell time within AOIs). The analyses followed an unbiased intent-to-treat (ITT) principle and included all participants who were randomized and started the intervention (if assigned to the treatment group). Finally, all parameter estimates in log odds (i.e., behavioral accuracy) were converted to odds ratio for effect size estimation.

Data analyses were conducted using R and the *lme4* package^34^ (R Core Team, 2020). The general strategy was to fit generalized mixed effects models with random effects for participants and stimulus item (categorical). Post-hoc comparisons from significant interactions were evaluated using the ‘*emmeans*’ R package and corrected for multiple comparisons using the False Discovery Rate procedure. Confidence Intervals were calculated using the Wald method.

First, we investigated whether dwell time to faces differentially increased as a function of participating in the intervention. In this analysis, dwell time to the face AOI was the dependent measure, group (intervention, control) and session (pre, post-intervention) were fixed factors. A treatment effect would be evident in a significant group x session interaction for *increasing* visual attention to the face over time.

Second, we evaluated the moderating effect of dwell time on task accuracy (correct = 1; incorrect = 0), in separate generalized linear mixed effects models (with a binomial link function). Specifically, we included the fixed factors of group (intervention, control), session (pre-, post-intervention), and dwell time (i.e., to faces or target objects). We fit separate models to evaluate how dwell time to faces and target objects differentially moderated accuracy over sessions and as a function of group using the following equations:

$$log\left( {\frac{{p_{ijk} }}{{1 - p_{ijk} }}} \right) = b_{0} + b_{1} \left( {dwell\; time_{ijk} } \right) + b_{2} \left( {session_{ijk} } \right) + b_{3} \left( {group_{ijk} } \right) + b_{4} \left( {dwell\; time_{ijk} \times session_{ijk} \times group_{ijk} } \right) + u_{0i} + u_{0j}$$

$$u_{0i} \sim N\left( {0, \sigma_{{u_{0} }}^{2} } \right)$$, for participant i = 1,…, I.

$u_{0j} \sim N\left( {0, \sigma_{{u_{0} }}^{2} } \right)$, for stimulus item j = 1,…, J.

### Ethics approval and consent to participate

All parents and/or older adolescents provided written consent, and younger adolescents provided written assent according to procedures approved by the Institutional Review Board (IRB) at Penn State University.

## Results

A total of 40 adolescents on the autism spectrum were enrolled and successfully randomized into the study. Two families were eligible for the study, but after completing the pre-intervention assessments, were not interested in starting the intervention or participating in the remainder of the study. Therefore, these participants were replaced after randomization. As a result, a total of 40 participants completed all aspects of the study (N = 20 Intervention). Table 1 reports the demographic characteristics of the sample as a function of group as well as the intervention outcomes for the intervention group.Table 2 reports the behavioral accuracy and dwell times to the relevant AOIs as a function of group and session.

**Table 1:** Sample characteristics and intervention outcomes.

	Control	Intervention
*n*	20	20
Age, mean (SD), mo	163.8 (31.9)	165.8 (33.7)
Gender, No. (% Male)	17 (85.0)	16 (80.0)
IQ, mean (SD)		
FSIQ	99.7 (13.4)	100.5 (16.6)
VIQ	98.0 (18.5)	97.0 (15.4)
PIQ	100.6 (10.8)	104.0 (16.0)
ADOS, mean (SD)		
Total	13.8 (4.2)	13.4 (5.0)
Social affect	10.6 (3.9)	9.6 (3.9)
Restricted and repetitive behavior	3.3 (1.7)	3.8 (1.6)
Intervention outcomes		
Total gameplay, mean (SD), hr	NA	17.8 (4.8)
Total time in gaze tasks, mean (SD), hr	NA	11.4 (3.9)

Cells include means (SD).

**Table 2:** Primary outcomes over time.

	Pre-intervention		Post-intervention	
	Control (n = 20)	Intervention (n = 22)	Control (n = 20)	Intervention (n = 20)
Behavioral data				
Accuracy	81.0 (10.4)	76.5 (12.7)	80.9 (10.4)	84.9 (12.4)
Eye tracking data				
Lost data (track loss time)	370 (320)	420 (410)	400 (270)	420 (480)
Task engagement (total dwell time)	2540 (740)	2590 (780)	2580 (630)	2600 (790)
Social visual attention (face dwell time)	920 (530)	960 (610)	1030 (440)	1050 (600)
Visual attention (target object dwell time)	450 (210)	410 (240)	490 (230)	400 (260)

Cells contain Mean (SD). Accuracy data are presented as aggregated % correct. Eye tracking data are presented in msec.

### Data quality

Prior to analyzing primary eye tracking outcome measures, we investigated whether data loss (i.e., eye tracking loss time), and task engagement (i.e., total dwell time to each stimulus for each participant) varied as a function of group (treatment, control) or session (pre-, post-intervention). For data loss, there were no significant main effects of group (*b* =  − 0.009, *se* = 0.026, 95% CI[− 0.062, 0.045], *p* = 0.741) or session (*b* =  − 0.003, *se* = 0.006, 95% CI[− 0.014, 0.008], *p* = 0.615), and no significant group x session interaction (*b* = 0.006, *se* = 0.011, 95% CI[-0.016, 0.028], *p* = 0.571). For task engagement, there were no main effects of group (*b* = 0.042, *se* = 0.221, 95% CI[− 0.406, 0.49], *p* = 0.851) or session (*b* = 0.027, *se* = 0.027, 95% CI[-0.027, 0.08], *p* = 0.325), and no significant group x session interaction (*b* =  − 0.03, *se* = 0.053, 95% CI[− 0.133, 0.073], *p* = 0.569). Together, these results indicate that neither the amount of data loss nor overall task engagement can explain group or session differences in visual attention.

### Question 1: did SAGA increase visual attention to faces?

We evaluated whether visual attention to faces improved as a function of participating in SAGA. Figure 1 shows the mean dwell time to faces for each group as a function of session (pre-, post-intervention). In contrast to predictions, the analysis revealed no significant *treatment effect* on dwell time to faces. Specifically, the group x session interaction was not significant (*b* =  − 0.02, *se* = 0.04, 95% CI[− 0.11, 0.06], *p* = 0.64). Also, there was no main effect of group (*b* = 0.03, *se* = 0.17, 95% CI[− 0.31, 0.36], *p* = 0.86) on dwell time to faces. However, there was a main effect of session (*b* = 0.11, *se* = 0.03, 95% CI[0.05, 0.17], *p* < 0.01), indicating that both the intervention and control groups looked longer at faces during the post- (*M* = 1.04, *SD* = 0.52) compared to the pre- (*M* = 0.94, *SD* = 0.57) intervention session. In sum, SAGA did not differentially increase social visual attention to faces; both the intervention and control groups evinced a small increase in dwell time to faces over sessions (~ 100 ms).

### Question 2: were SAGA-related improvements in understanding eye gaze cues moderated by visual attention to *faces*?

**Figure 2:**
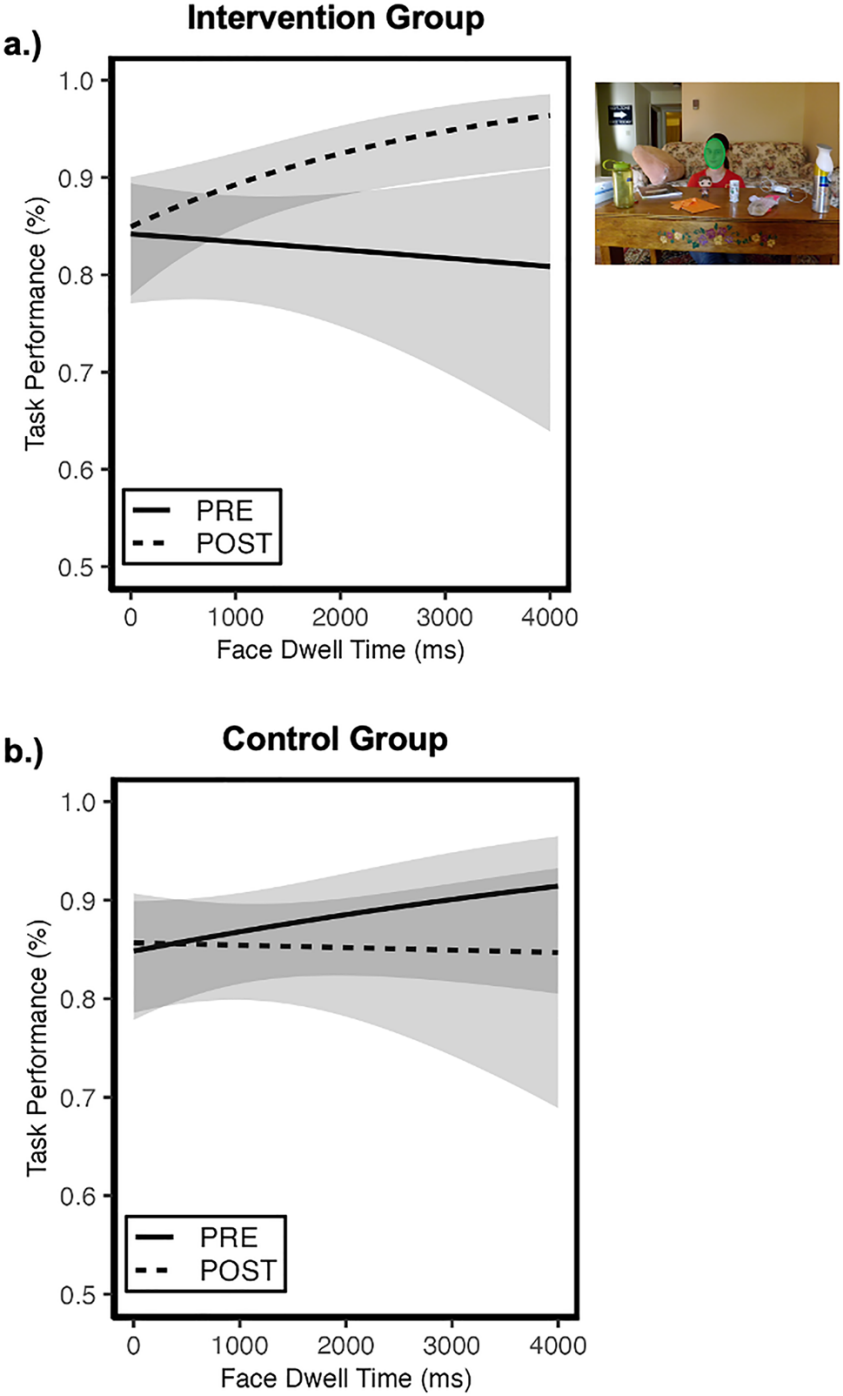
Change in association between social visual attention to faces and performance in the Gaze Perception task. The probability of identifying the correct target object is plotted as a function of dwell time to the *actor’s face* for each group separately over time. (**a**). Among the *intervention participants*, there was significant session x dwell time interaction. The follow-up analyses revealed that visual attention to faces (green area in example stimulus) did not predict understanding of directed gaze cues prior to the intervention (**a—**solid line). However, after participating in SAGA, the intervention participants were more likely to interpret the directed gaze and correctly identify target gazed-at objects when they looked longer at faces (**a**—dashed line). In contrast, for the *control group*, there was no session x dwell time interaction; visual attention to faces did not predict performance prior to (**b**—solid line) or 2-months later at the post-test (**b**—dashed line). Shaded region reflects 95% confidence intervals. All plots reflect model-predicted relations based on the mixed-effects models.

Figure 2 plots accuracy to interpret directed gaze cues as a function of dwell time to faces for each group and session. The generalized LMM revealed a significant group × session × dwell time interaction on task performance (*OR* = 1.87, *se* = 0.47, 95% CI[1.14, 3.08], *p* = 0.01). To interpret this 3-way interaction, we investigated potential session x dwell time interactions separately in the treatment and standard care control groups.

In the treatment group (Fig. 2a), we observed a significant session x dwell time interaction (*OR* = 1.56, *se* = 0.28, 95% CI[1.11, 2.21], *p* = 0.01). To interpret this 2-way interaction we conducted separate analyses of the main effect of dwell time within each session. At the pre-intervention session, there was no main effect of dwell time on accuracy in the treatment group (*OR* = 0.94, *se* = 0.12, 95% CI[0.73, 1.22], *p* = 0.66). In other words, visual attention to faces did not predict understanding of directed gaze cues prior to the intervention (see Fig. 2a). However, at the post-intervention session, there was a significant main effect of dwell time to faces (*OR* = 1.47, *se* = 0.21, 95% CI[1.11, 1.95], *p* < 0.01), indicating that when treatment participants looked longer at faces, they were more likely to interpret the directed gaze and correctly identify target gazed-at objects (see Fig. 2a).

We did not find the same pattern of results in the standard care control group (see Fig. 2b). The session x dwell time interaction was not significant (*OR* = 0.84, *se* = 0.16, 95% CI[0.58, 1.20], *p* = 0.33). Follow up analyses confirmed that there were no main effects of dwell time on accuracy during either the pre-intervention (*OR* = 1.17, *se* = 0.17, 95% CI[0.89, 1.55], *p* = 0.25) or post-intervention (*OR* = 0.98, *se* = 0.14, 95% CI[0.74, 1.30], *p* = 0.89) sessions. These findings reveal that social visual attention to faces did not predict referential understanding of gaze for the control group in either session.

### Question 3: were SAGA-related improvements in understanding of eye gaze cues moderated by visual attention to *target objects*?

**Figure 3:**
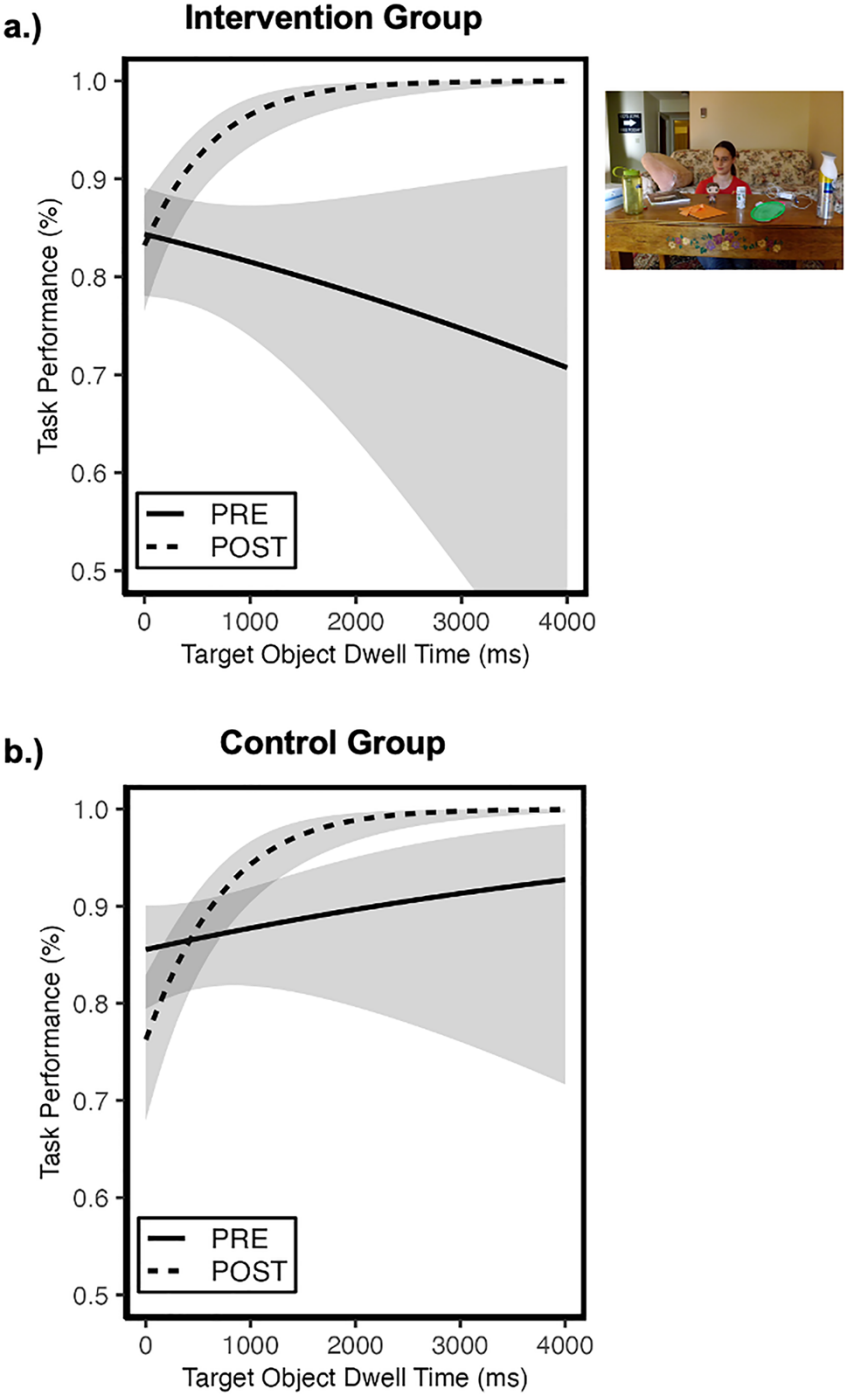
Association between visual attention to target objects and performance in Gaze Perception task. The probability of identifying the correct target object is plotted as a function of dwell time to the *target object* (green area in example stimulus) for the treatment (**a**) and control (**b**) groups separately. For both groups, dwell time to target objects did not predict performance prior to the intervention (solid lines). However, by the post-intervention session, longer dwell times to target objects were associated with improved task performance for both groups of adolescents (dashed lines). Shaded region reflects 95% confidence intervals. All plots reflect model-predicted relations based on the mixed-effects models.

We also evaluated whether the improvements in sensitivity to eye gaze cues were moderated by visual attention to target gazed-at objects. Figure 3 plots accuracy to interpret directed gaze cues as a function of dwell time to target objects for each group and session.

The generalized LMM failed to reveal a significant group × session × dwell time interaction on accuracy (*OR* = 1.62, *se* = 0.88, 95% CI[0.56, 4.67], *p* = 0.38). However, there was a significant session x dwell time interaction (*OR* = 5.42, *se* = 1.51, 95% CI[3.14, 9.36], *p* < 0.01). To interpret this two-way interaction, we investigated the potential main effect of dwell time on accuracy for each session separately (collapsed on group). During the pre-intervention session, there was no main effect of dwell time (*OR* = 0.99, *se* = 0.16, 95% CI[0.74, 1.35], *p* = 0.97). However, a significant main effect of dwell time emerged at the post-intervention session (*OR* = 5.40, *se* = 1.30, 95% CI[3.37, 8.65], *p* < 0.01). As shown in Fig. 3, both groups showed a strong positive association between dwell time to target objects and accuracy post-intervention. The nature of this relationship was not different between the standard care control and treatment groups. Therefore, this emerging association between visual attention to *gazed-at target objects* and the ability to interpret shifts in gaze was not related to the intervention treatment. Instead, it may reflect a learning-related practice effect or a more generalized developmental improvement. The full set of results relating behavioral performance and social visual attention are represented in Table 3.

**Table 3:** Summary of results linking social visual attention and behavioral performance in eye Gaze task.

	Pre-intervention session		Post-intervention session	
	Faces	Target objects	Faces	Target objects
Autistic intervention	No association	No association	Longer SVA = > higher accuracy	Longer SVA = > higher accuracy
Autistic control	No association	No association	No association	Longer SVA = > higher accuracy
Non-autistic control^11^	Shorter SVA = > higher accuracy	Longer SVA = > higher accuracy	NA	NA

*SVA* Social visual attention—measured via eye tracking technology.

## Discussion

SAGA was developed using the experimental therapeutics approach with the goal of improving sensitivity to eye gaze cues as a tool of non-verbal social communication for autistic adolescents. In prior work, we demonstrated the initial effectiveness of SAGA to improve referential understanding of eye gaze cues among autistic adolescents^17^. Specifically, autistic adolescents who completed the SAGA intervention improved their understanding of eye gaze cues as tools for nonverbal social communication. They were more accurate at perceiving and interpreting the communicative intent (i.e., to look at the target object) of directed gaze in human faces, even though the game trained them on avatar faces.

In the present work, we investigated whether this improved behavioral sensitivity to eye gaze cues was facilitated by *increasing visual attention to faces and/or to the target objects* of the directed gaze. SAGA is designed to implicitly scaffold social attention to faces. Importantly, there are no explicit instructions to look at avatar faces; the intervention game only provides opportunities for learning about how nonverbal social cues (e.g., directed shifts in eye gaze) guide and contextualize social interactions in the game. As a result, it is important to evaluate whether improvements in the ability to perceive and interpret these nonverbal social cues are moderated by changes in social visual attention. Here, we predicted that if social visual attention is an underlying mechanism facilitating the improvement of sensitivity to eye gaze cues in the SAGA intervention, we would expect to see increased attention to faces overall, which would predict better ability to interpret eye gaze cues, specifically in the treatment group.

### Did SAGA increase social visual attention to faces?

Prior to the SAGA intervention, the participants in both groups attended to faces for approximately 24% of the total duration of the 4000 ms trials. They were not different in this measure of social visual attention to faces. In contrast to predictions, the treatment group did not exhibit a disproportionate increase in visual attention to faces following the intervention. Instead, both groups of autistic adolescents increased dwell time to faces over sessions. The increase amounted to ~ 100 ms, which was a small effect. This increase in social visual attention to faces from *both* groups existed despite the finding that only the treatment group exhibited an improvement in behavioral performance on the same task^17^. Therefore, this general increase in social visual attention to faces is not related to the effects of the intervention. It may reflect a practice effect in the context of repeatedly performing the Gaze Perception task. It is important to note that a small portion of the stimuli (37.5%) in this task were consistent across sessions. Therefore, the magnitude of this practice effect might be even larger if more of the stimuli were the same in both testing sessions. In sum, the treatment group exhibited an increase in social visual attention to faces and an improvement in the ability to interpret the eye gaze cues in the task, while the control group evinced an increase in social visual attention to faces in the absence of an improvement in the ability to identify the gazed-at target object. One interpretation of these findings is that repeatedly engaging in the Gaze Perception task increases social visual attention to faces but does not improve referential understanding of eye gaze cues.

### Were SAGA-related improvements in understanding eye gaze cues moderated by visual attention to *faces*?

Given that the treatment group exhibited both an increase in social visual attention to faces and an improvement in the ability to understand eye gaze cues, we were interested in understanding whether these outcomes were fundamentally related. To evaluate this question, we assessed whether attention to faces predicted performance in the Gaze Perception task differentially for each group over time.

Prior to the SAGA intervention, attention to faces failed to predict performance in the Gaze Perception task for both the treatment and standard care control groups. In other words, longer dwell times to faces did not predict the ability to identity the gazed-at target object during the pre-intervention testing session. However, following the SAGA intervention, an association emerged between attention to faces and the ability to accurately identify the target-object of directed gaze shift for the *treatment group*. Specifically, following participation in the SAGA intervention, when autistic adolescents looked longer at faces, they were more likely to correctly interpret the gaze cues and identify the target gazed-at object. This emerging association between social visual attention and performance in the Gaze Perception task was not evident in the standard care control group. Therefore, increasing social visual attention to faces does appear to moderate SAGA-related improvements in the ability to interpret and understand directed gaze as a non-verbal social communicative cue in autism.

There are two important points to consider regarding this finding. First, the modulatory effect of social visual attention on sensitivity to process directional eye gaze cues in the treatment group is not explained by differential quality of data or task engagement between groups. These metrics were comparable across groups. Second, the modulatory effect in the treatment group does not simply reflect test–retest improvements in performance. Most stimuli (~60%) were novel across sessions (to test for generalization of learning) and the standard care control group did not improve in the ability to use gaze cues to identify the target object at the post-intervention session. This suggests that only the treatment group exhibited generalization of learning.

Therefore, the SAGA intervention appears to help develop sensitivity to the role of directed gaze in non-verbal social communication, in part, by facilitating attention to faces. Visual attention to faces is critical so that the trajectory of gaze can be computed, and the referential understanding of gaze can be discovered. Currently, it is unclear whether SAGA improves social visual attention to faces in the context of other non-verbal social communicative tasks (e.g., interpreting emotional expressions). This is an important test of generalization of learning that will need to be evaluated in future work.

It is interesting to consider the performance of the treatment group following the intervention in relation to another control group, non-autistic adolescents who performed the Gaze Perception task and did not undergo the intervention training^11^. Indeed, non-autistic adolescents also exhibit a relation between social visual attention to faces and the ability to identify target gazed-at objects in the context of performing the Gaze Perception task. However, the non-autistic adolescents exhibit a very different pattern than do the autistic adolescents who completed the SAGA intervention. During the Gaze Perception task, non-autistic individuals with the *shortest* looking times to faces tended to be the most successful at identifying the target object in the task^11^. We suggested that this finding could reflect increasingly efficient perception of gaze and computation of gaze trajectory during adolescence^11^. When thinking about all the findings together from non-autistic adolescents, autistic control participants, and autistic treatment participants in the Gaze Perception task, an interesting potential interpretation emerges. This difference in findings might reflect *differential stages of learning* about how “eyes are meaningful.” In other words, the non-autistic adolescents may have a longer developmental history of attending to, perceiving, processing, and understanding eye gaze cues. Therefore, the relatively shorter attention to faces when they exhibit correct performance may reflect a fine-tuning and increasing efficiency of gaze processing.

The behavior of autistic adolescents with no SAGA training may reflect a state in which there have been limited opportunities for *learning* about how shifts in eye gaze can function as cues for social communication. SAGA may provide an opportunity to catch up quickly in this learning process because of the way social visual attention and sensitivity to eye gaze cues are scaffolded in a clear, calm, safe environment. As a result, the autistic adolescents with SAGA treatment appear to have begun the learning process about how to compute gaze trajectory and/or determine the referential intent of directed gaze. Therefore, accurate processing of this information may take time (i.e., require longer attention to faces) and practice to learn the parameters and predictors of the cues in the real world.

It is still an open question whether SAGA provided autistic adolescents with the experiences and/or perceptual tools to learn about eye gaze in the real world in ways that generate similar processing strategies used by non-autistic adolescents. It will be important to investigate the underlying nature of the longer-term perceptual processing strategies that autistic adolescents derive for gaze processing following SAGA treatment, which was developed based on models of early learning differences in the social world of autistic individuals. For example, additional follow-up testing will help investigate the hypothesis that the emerging relation between longer looking times to faces and better understanding of eye gaze cues in the autistic treatment group reflects an earlier stage of the developmental processes of learning to understand and process eye gaze cues as tools of nonverbal social communication.

### Visual attention to target objects and understanding directed gaze

As with social visual attention to faces, visual attention to target objects did not predict performance in the Gaze Perception task for either group of autistic adolescents during the pre-intervention session. In other words, as with attention to faces, attention to target objects was not associated with the ability to correctly identify the gazed-at target object in the task. However, during the post-intervention session, *both groups* of autistic adolescents (treatment and standard care control) exhibited a different pattern. For both groups of participants, longer attention to target objects predicted more accurate performance. That is, at the post-intervention session, participants were more likely to correctly interpret the referential intent of directed gaze when they attended *longer* to target objects. Importantly, because this pattern emerged in both groups of participants, it is not related to participation in the SAGA intervention. Instead, it appears to be a learning-related effect that is facilitated by engaging with the Gaze Perception task.

There are multiple issues to consider in the context of this finding as well. First the task instructions were to “Identify the object that the person is looking at.” These directions explicitly associate eye gaze with objects in the world. In so doing, the instructions may provide a functional link between directed gaze and referential intent in a task environment that supports learning this association. A test of this hypothesis could involve assessing performance in this task with a different set of instructions that do not link eye gaze and referential intent (i.e., the free condition^9^)—like, “Look at the actor. Identify what they are doing.” These instructions draw attention to the actor and the act of seeing, but no longer infer referential intent of looking at a specific object in the world.

Second, although both groups exhibited a new pattern of behavior at the post-intervention session in which longer attention to target objects predicted more accurate performance, autistic adolescents in the standard care control group still perform worse than autistic adolescents in the treatment group. In other words, the emerging relation between visual attention to target objects and performance in this task is not sufficient to enable the same level of performance acquired by the treatment group. Therefore, it appears that more attention to both target objects and faces predicts the improved performance in the treatment group.

Third, the emerging relation between increasing visual attention to target objects and task performance is in line with the pattern of behavior observed for non-autistic adolescent behavior in this Gaze Perception task^11^. When non-autistic adolescents attend longer to target objects, they are also more likely to correctly identify them in this task. So, on one hand it appears that engaging with the Gaze Perception task leads to modulation of visual attention to the gazed-at target objects in autistic adolescents that is more consistent with the behavior of non-autistic adolescents in this task. However, when all the results are considered together, it becomes clear that the treatment group’s behavior is still different from that of their non-autistic peers (see Table 3).

Specifically, among non-autistic adolescents both longer attention to target objects together with shorter attention to faces predict the ability to correctly interpret gaze cues and identify the target objects in the Gaze Perception task^11^. The autistic adolescents in the standard care control group develop a pattern in which only longer attention to target objects predicts better performance in the task. Their social visual attention to faces is unrelated to their task performance. Among the ASD treatment group, when individuals attend longer to faces and longer to target objects, they are more likely to exhibit accurate performance in the task. One prediction is that with more practice honing their skills interpreting eye gaze cues, the intervention participants will advance learning about these processes and ultimately begin to show even more similar patterns of learning reflected by the non-autistic adolescents (i.e., less attention to faces predicting better performance). Alternatively, the longer-term performance of the intervention participants may reflect a different strategy for using social visual attention to processing eye gaze information. Either set of findings has the potential to improve and augment existing models of social visual attention in ASD by demonstrating how learning influences social visual attention once it has been engaged.

### Limitations and future directions

This evaluation of the effectiveness of SAGA is embedded in a larger program of research that aims to evaluate the clinical relevance and generalization of learning to real world social interactions in autistic adolescents. Our goal in this study was to determine the feasibility of social visual attention as a target mechanism underlying improvement in sensitivity to eye gaze cues resulting from participation in the SAGA intervention. Going forward, it will be important to test SAGA against an appropriately matched control game and in a larger sample to provide converging and more rigorous evidence of its effectiveness. In so doing, it will be important to employ double-blinding procedures in an RCT. Also, it will be important to evaluate how far the learning generalizes to improve untrained behaviors, including dynamic social-communicative interactions, and clinical symptoms. Future work is needed to evaluate the persistence of these improvements and to understand whether there are individual differences that impact the effectiveness of the intervention (e.g., gender, age). Finally, it is essential to evaluate the longer-term effects of SAGA training on the lived experiences of autistic individuals to understand whether and how it contributes in positive and/or negative ways to their lives.

## Conclusion

We present converging findings that support the effectiveness of SAGA as a psychosocial intervention to improve sensitivity to and understanding of eye gaze cues as a tool of non-verbal social communication in autistic adolescents. Critically, we show that social visual attention to faces moderates the behavioral treatment response among intervention participants. There is much interest in measuring social visual attention as a biomarker for determining risk and treatment responses in ASD^35,36^. To our knowledge, this is the first study to measure social visual attention via eye tracking as a *treatment response* in a *clinical trial* of a psychosocial intervention for autism. Notably, we report that social visual attention in absence of behavioral data (as in a passive viewing task) does not predict treatment responses. Our results suggest that it is essential to assess how social visual attention is deployed in the service of *non-verbal social communication* when measuring it as a treatment response. An implication of these results is that that once social visual attention it engaged in autism, learning may impact the way it is deployed to support non-verbal social communication over time.

## Data Availability

The data that support the findings of this study will be uploaded to the National Institutes of Health National Data Archive (NDA) https://nda.nih.gov. The stimuli are available in Databrary http://doi.org/10.17910/b7.884.
